# MicroRNA in localized scleroderma: a review of literature

**DOI:** 10.1007/s00403-019-01991-0

**Published:** 2019-10-21

**Authors:** Katarzyna Wolska-Gawron, Joanna Bartosińska, Dorota Krasowska

**Affiliations:** Chair and Department of Dermatology, Venerology and Paediatric Dermatology, Medical University of Lublin, Lublin, Poland

**Keywords:** Localized scleroderma, Morphea, LoSC, MicroRNA, miRNA

## Abstract

Localized scleroderma (LoSc) is rare connective tissue disease that manifests with inflammation and fibrosis of the skin. Depending on the LoSc subtype, adjacent structures such as subcutaneous tissue, fascia, muscles, bones may be affected. The hallmark of fibrosis is tissue remodelling with excess deposition of extracellular matrix proteins (ECM), principally collagens. MicroRNAs (miRNAs) are small, noncoding RNA molecules that consist of 19–24 nucleotides and act as negative regulators of gene expression at the posttranscriptional level. Based on the current articles, approximately 40 microRNAs have been linked to fibrosis in different organs and diseases. The majority of these molecules promote or inhibit fibrosis by targeting connective tissue growth factor (CTGF), extracellular matrix proteins, TGF-β pathway and MAPK (mitogen-activated protein kinase) pathway. Further, particular microRNAs regulate fibrogenesis by altering epithelial-to-mesenchymal transition (EMT) or activating proliferation of myofibroblasts. MiRNAs are relatively stable, detectable in tissues and body fluids (serum, plasma) which suggest that they may serve as beneficial biomarkers to monitor the course of the disease and response to treatment. Herein, we report the present state of knowledge on microRNA expression in localized scleroderma.

## Localized scleroderma

**Fig. 1:**
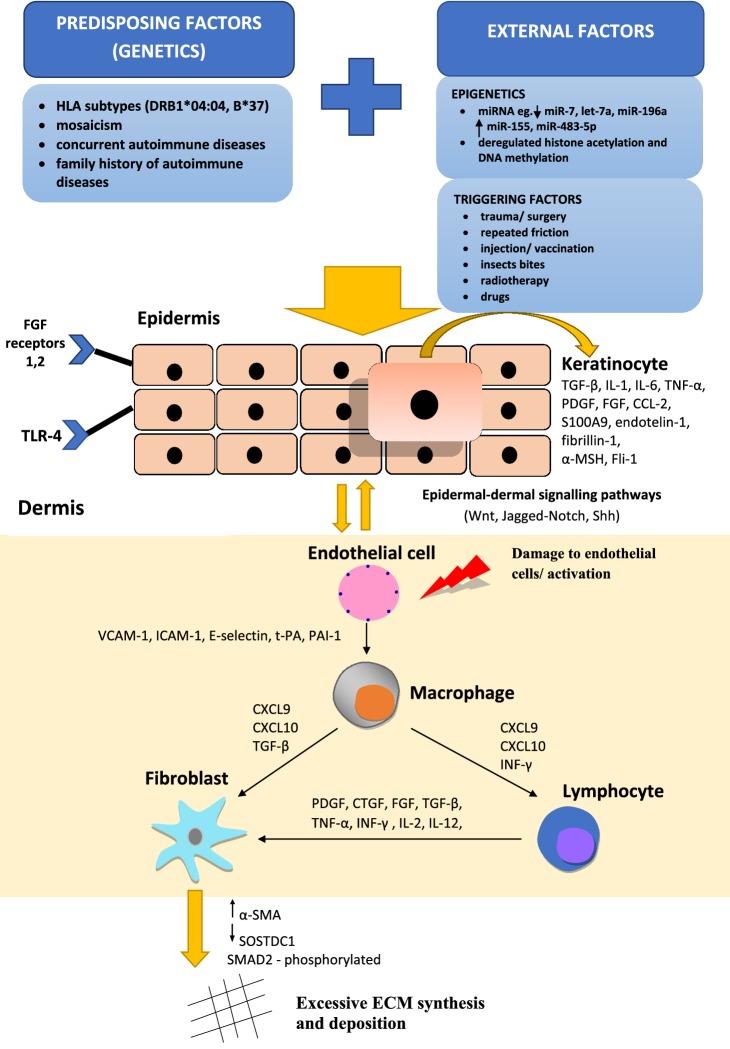
The etiopathogenesis of localized scleroderma [3, 26, 30]

Localized scleroderma is a rare, autoimmune disease that affects dermis and sometimes structures lying beneath the skin. The incidence rate is reported to range from 0.4 to 2.7 cases per 100 000 people [14]. Females are more frequently affected then men (2.6–6 times) [15]. According to German classification, localized scleroderma may be divided into five clinical subtypes—limited, generalized, linear, deep and mixed [14, 15]. Circumscribed morphea is the most common type that usually affects adults between 40 and 50 years of age [15, 20]. Linear LoSc is frequently present in children aged 2–14 [15]. The disease outcome relies on the subtype of localized scleroderma and extent of skin lesions. Delayed diagnosis may lead to time lapse in therapy and, in consequence, functional disabilities and disfiguration [14, 15]. The etiopathogenesis of localized scleroderma has not been fully understood yet, however, a number of immunoinflammatory and profibrotic factors are likely implicated (Fig. 1) [3, 26, 30]. Evidence points to LoSc as an autoimmune disorder due to family/personal history of autoimmune diseases, presence of certain human leucocyte antigen (HLA) subtypes and high prevalence of autoantibodies. 10–30% of patients with localized scleroderma reported having a family history of autoimmune disease, whereas 10% of them present with a concomitant autoimmune disorder [especially vitiligo, alopecia areata, rheumatoid arthritis (RA)] [30]. Studies examining HLA association with LoSc revealed that HLA-B*37 and HLA-DRB1*04:04 lead to increased susceptibility, especially in linear and generalized subtypes of LoSc [26, 30]. Additionally, HLA-DRB1*04:04 strongly correlates with increased risk for rheumatoid arthritis what implies a common genetic susceptibility to LoSc and RA [30]. Cases of linear localized scleroderma, manifesting with skin lesions following Blaschko’s lines, confirm a potential relevance of mosaicism [26, 30]. Epigenetic mechanisms may stand for a feasible link between genetic and external (environmental) factors [3, 26]. Over the last decade, microRNAs have attracted tremendous interest as a pivotal epigenetic regulators in LoSC. Additionally, deregulated histone acetylation and DNA methylation have been demonstrated in localized scleroderma [26].

A number of external factors promote diseases onset in susceptible patients. Injuries, repeated friction (especially along with bra straps, waistline, groins), injections/vaccinations (hepatitis B, tetanus, vitamin B12), insect bites may induce localized scleroderma in about 16% of adults and 9% of children [26]. Postirradiation (notably radiotherapy of breast cancer) and drug-induced (ex. bisoprolol, bleomycin, d-penicillamine, anti-TNF-α) localized scleroderma have also been reported [26].

Triggering event in a susceptible person leads to activation of keretinocytes and production of many factors driving fibrosis such as transforming growth factor β (TGF-β), tumor necrosis factor-α (TNF-α), platelet-derived growth factor (PDGF), fibroblast growth factor (FGF), friend leukaemia integrated transcription factor (Fli-1), endotelin-1 (ET-1), fibrillin-1, α-melanocyte-stimulating hormone (α-MSH), interleukin 1 (IL-1), IL-6, S100A9 [26]. Production of protein S100A9 occurs further to epidermal stress and via toll-like receptor 4 (TLR-4) induce fibroblast proliferation by potentiating TGF-β activity [26]. Additionally, knocking down FGF receptors 1 and 2 results in activation of keratinocytes and release of profibrotic factors (S100A9, IL-1 [26]). Keratinocytes interact with endothelial cells and fibroblasts through signalling pathways including Wnt, Sonic hedgehog (Shh) and Jagged-Notch [26].

Damage to endothelial cells due to infection, inflammation, autoimmune reaction or skin injury leads to the secretion of cytokines and adhesion molecules (intracellular adhesion molecule-1 (ICAM-1), vascular cell adhesion molecule-1 (VCAM-1), E-selectin, CXCL-8), resulting in the recruitment of immune cells (lymphocytes, macrophages) [3, 27, 29]. These immune cells produce principal fibrotic growth factors such as transforming growth factor beta (TGF-β), connective tissue growth factor (CTGF) and platelet-derived growth factor (PDGF), driving fibroblast activation, proliferation and differentiation [3, 27, 31]. Badshah and co-workers have recently reported that fibroblasts exhibit continuous activation of phosphorylated SMAD2 which is important in TGF-β signalling pathway [4]. Moreover, they shed light on upregulation of α-SMA (α-smooth muscle actin) and downregulation of SOSTDC1 (sclerostin domain containing 1—antagonist of TGF-β and Wnt signalling pathways) in these cells [4]. Both persistent fibroblast proliferation and differentiation into myofibroblasts results in excessive extracellular matrix (ECM) synthesis and deposition [3, 27]. Myofibroblasts arise from a variety of sources including circulating fibroblasts, resident mesenchymal cells, epithelial and endothelial cells in processes defined as epithelial-to-mesenchymal transition (EMT) [3, 27].

## MicroRNA

**Fig. 2:**
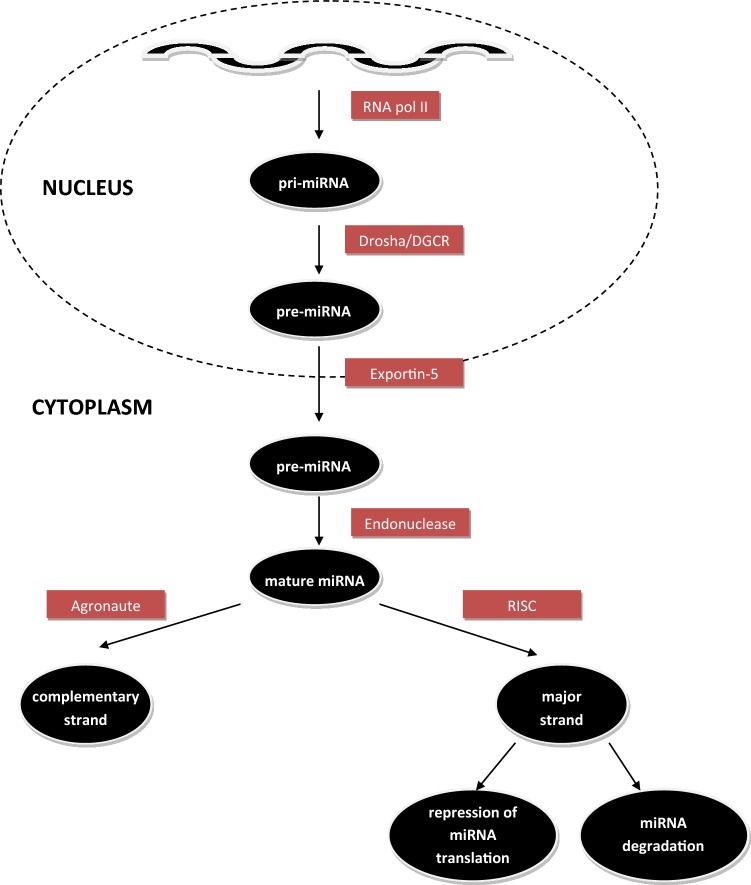
The biogenesis and maturation of microRNAs [3, 26]

MicroRNAs belong to small, noncoding RNAs that represent crucial regulators of cell differentiation, proliferation, apoptosis and immune response [25, 28]. Thus far, according to miRBase, 1917 microRNAs encoding sequences have been determined in the human genome. The biogenesis and maturation of microRNAs take place in several steps (Fig. 2). Synthesis of miRNA starts within nucleus where RNA polymerase II transcribes genes encoding miRNA to form primary microRNA (pri-microRNA)—a steam loop structure that is composed of several hundred to few thousands nucleotides [13, 28]. The primary transcript is cleaved by microprocessor complex (RNase III enzyme Drosha and DGCR8) into premature miRNA (pre-microRNA) that consists of 60–70 nucleotides [5, 8]. Pre-microRNA is then translocated into the cytoplasm by exportin-5 (XPO5) and further processed by endonuclease Dicer into mature double-stranded microRNA (22 nucleotides long) [3, 28]. One strand (passenger stands/complementary stand) is destructed by Argonaute proteins, the other strand (guide strand/mature strand) associates with RISC (RNA-induced silencing complex). The mature miRNA interact with 3′untranslated region (UTR) of target mRNA by one of two mechanisms of gene regulation—mRNA degradation or repression of mRNA translation, depending on the degree of complementarity of microRNA and mRNA [13, 28]. Compelling complementarity lead to mRNA degradation, whereas confined complementarity guide repression of mRNA translation [3, 28].

MicroRNAs are now considered to play a pivotal role in the regulation of certain processes related to the development of number human diseases such as cancers, cardiovascular, autoimmunologic, neurodegenerative, liver and skin diseases [1, 23]. Among dermatologic ones, psoriasis, atopic dermatitis, allergic contact dermatitis, lichen planus, localized and systemic scleroderma, bullous diseases, alopecia, vitiligo and skin cancers have been established [1, 23].

## MicroRNA and fibrosis

Recent reports have shown that several microRNAs participate in regulation of processes that drive fibrosis, including transforming growth factor (TGF-β) signalling, fibroblast proliferation and differentiation, extracellular matrix proteins deposition and epithelial-to-mesenchymal transition (EMT) [3, 18, 34]. Upregulation of some profibrotic microRNAs results in fibrosis, whereas antifibrotic miRNAs suppress this process and may be knock-down in fibrosis [13].

According to literature, MiRNAs involved in TGF-β signalling cascade include miRNA-18, miRNA-20, miRNA-21, miRNA-23b, miRNA-29, miRNA-140-5p, miRNA-146a, miRNA-206 [3, 16]. MiRNA supposed to regulate fibroblast proliferation and differentiation are miRNA-21, miRNA-31, miRNA-146a and miRNA-200 family [3, 16]. Molecules affecting extracellular matrix synthesis and deposition are miRNA-let-7a, miRNA-7, miRNA-26a, miRNA-29, miRNA-129-5p, miRNA-133a, miRNA-133b, miRNA-150, miRNA-196a [3].

## Materials and methods

A review of the literature published from January 2000 to October 2018 was performed using MEDLINE, Scopus, Web of Science, Clinical Key databases according to PRISMA (Preferred Reporting Items of Systematic Reviews and Meta-Analyses) guidelines. A search of databases was conducted utilizing MeSH terms: “localized scleroderma” OR “morphea” OR “circumscribed scleroderma” OR “dermatosclerosis” AND “microRNA” OR “micro RNA” OR “miRNA” OR “miRNAs”.

## Results

**Fig. 3:**
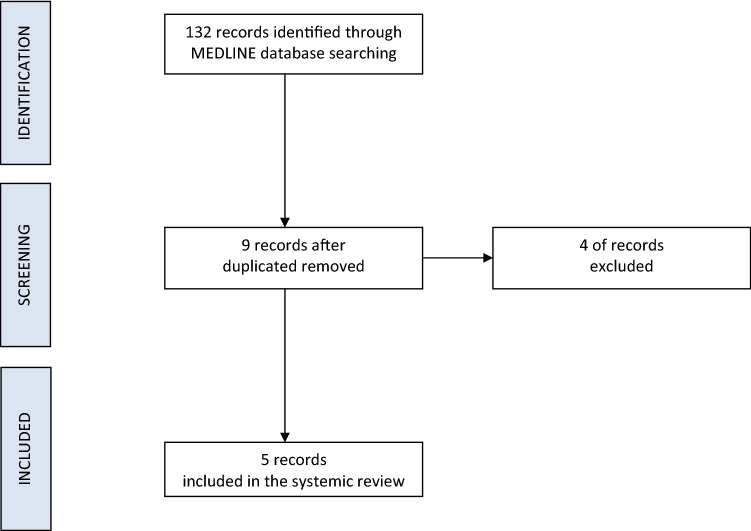
Literature searched based on PRISMA protocol

The preliminary investigation revealed 132 articles (Fig. 3). After duplicates were removed, a total of 9 results were screened. Of the remaining records, 1 was irrelevant to localized scleroderma and 3 were review articles on systemic scleroderma. This resulted in 5 clinical studies that met inclusion criteria and were included in this review (Table 1).

**Table 1:** Studies included in this systemic review [6, 8, 16, 17, 29]

Study	Etoh et al. 2013 [8]	Makino et al. 2013 [16]	Makino et al. 2014 [17]	Yan et al. 2016 [29]	Chouri et al. 2018 [6]
Study group (LoSc patients)	30/5	32/7	34/3	7	22
No of patients with circumscribed LoSc	15	19	No data	No data	No data
No of patients with liner LoSc	10	8	No data	No data	No data
No of patients with generalized LoSc	5	5	No data	No data	No data
Control group	20/7	17/7	22/3	9	9
Material	Serum/biopsy	Serum/biopsy	Serum/biopsy	Biopsy	Serum
miRNA	miRNA-7	miRNA-let7a	miRNA-196a	miRNA-155	miRNA-483-5p
Regulation	Downregulation	Downregulation	Downregulation	Upregulation	Upregulation

### MicroRNA-7

The role of miRNA-7 in the function of the skin remains unexplained, however, modified miR-7 expression has been stated in wound healing process and fibrotic skin diseases (scleroderma, dermatomyositis) [11]. Etoh and co-workers reported downregulated levels of miRNA-7 in 30 serum samples of localized scleroderma patients as well as skin biopsies of 5 patients with LoSc and 3 patients with keloid [9]. Among localized scleroderma patients, mean miR-7 levels were lower in individuals with linear type of LoSc, comparing those with morphea or generalized LoSc [9]. Serum microRNA-7 concentration was inversely correlated with disease duration and mLoSSI (Localized Scleroderma Skin Activity Index), but not significant [9]. Knocking down of microRNA-7 in normal fibroblasts caused overexpression of COL1A2 which was demonstrated to be a direct target of miR-7 [11, 25]. Therefore, miR-7 may carry in pathogenesis of localized scleroderma due to overexpression of type II collagen. Serum miR-7 can be considered as a promising biomarker and modulation of miR-7 in dermal fibroblast may be favourable method of treatment of LoSc. [8, 21].

### miRNA-let 7a

The let-7 family miRNAs were the first discovered human microRNAs [12, 24]. MicroRNA-let 7a is another molecule that is knockdowned in both serum and dermal fibroblast of LoSc patients. Makino and collaborators demonstrated that miR-let 7a levels in 7 localized scleroderma skin biopsies and 7 systemic scleroderma (SSc) skin samples were significantly decreased, comparing with 7 healthy skin biopsies and 5 keloid skin samples [17]. The decline of miRNA-let 7a concentration in LoSc skin samples was greater that in systemic scleroderma ones [17]. Serum miRNA-let 7a levels in patients with localized scleroderma were remarkably lower than those in the control cohort which is in accordance with the reduction of miRNA let-7a in LoSc skin [17]. Furthermore, there was no prominent difference in the levels of miRNA-let 7a among the three groups of localized scleroderma (19 morphea, 8 linear, 5 generalized) [17]. Authors reported no correlation between the serum level of microRNA-let 7a and following parameters: number of lesions, disease duration, soluable IL-2R and ss-DNA [17]. Effectiveness of miRNA-let 7a mimics was successfully verified in a BALB/cAJcl mouse model of bleomycin-induced skin fibrosis [17]. Let 7a oligonucleotides were injected transdermally into the shaved back of mice which caused increase in let-7a concentration in the skin and linked improvement in skin fibrosis [3, 17]. Authors attempted to elucidate the mechanism that mediates reduced expression of microRNA-let 7a in scleroderma fibroblasts [17]. They stimulated healthy fibroblasts with exogenous TGF-β1 which guided reduced microRNA-let 7a expression [17].

### miRNA-196a

MicroRNA-196a is a putative regulator of α1(I) and α2(I) chains that are components of type I collagen. Makino and co-workers reported that depletion of miRNA-196a level in 3 localized scleroderma skin biopsies was statistically significant compared with 3 keloid skin samples and 3 normal skin biopsies [18]. Consistent with knockdown of microRNA-196a in LoSc skin, the serum concentrations of miRNA-196a were remarkably diminished in patients with localized scleroderma (34) in contrast to healthy controls (22) [18]. Nonetheless, there was no correlation between serum levels of miRNA-196a and types of LoSc, clinical manifestations or laboratory tests. In cultured dermal fibroblasts, knockdown of miRNA-196 was normalized by depletion of TGF-β level [18, 25]. Inhibition of microRNA-196a enhanced type I collagen synthesis in normal fibroblast, while transfection of the microRNA-196 mimic lead to downregulation of collagen in scleroderma fibroblast [11, 18, 25]. Two years earlier, the same authors reported that in systemic scleroderma there was no prominent variation between healthy controls and patient group [10]. Divergences between systemic and localized scleroderma may be caused by different extension of skin fibrosis [18]. LoSc lesions tend to be more severe, affecting deeper structures lying beneath the skin (subcutaneous tissue, fascia, muscles, bones) in contrast to systemic scleroderma lesions [18].

### miRNA-155

MicroRNA-155 is a profibrotic molecule that has been identified to regulate endothelial-to-mesenchymal transition (EndoMT)—differentiation by which endothelial cells (ECs) lose their specific structure/junctions and acquire myofibroblast-like features [6, 19]. Moreover, miRNA-155 is a pivotal, negative regulator of the inflammatory response of the pattern recognition receptors (toll-like receptors, TLR) [22]. One of the foremost ligands for TRL4 is lipopolysaccharide (LPS) which has been reported to alter the expression level of miR-155 [22]. Yan and co-workers demonstrated that miRNA-155 expression was upregulated in scleroderma patients’ skin (19) as opposed to control group (9) [33]. Furthermore, the expression of miRNA-155 was higher in localized scleroderma samples (9) comparing with systemic scleroderma ones (12) [33]. Authors showed that miRNA-155 expression in the lesional skin correlated positively with the expansion of skin involvement in SSc patients [33]. Yan and collaborators found that miRNA-155 regulated Akt and Wnt/β-cathenin pathways [2, 33]. MiRNA-155 mimics firmly diminished the degradation of β-cathenin and increased the phosphorylation of Akt, whereas miRNA-155 inhibitor acted inversely to above pathways [2, 33]. The study revealed an intending, innovating treatment approach to target miRNA-155 [33]. Two weeks after bleomycin-induced skin fibrosis in mice models, topical anagomiR-155 was applied, revealing significant depletion of dermal thickening and collagen desposition [33]. These promising results imply that epicutaneous antagonist of miRNA-155 could be favourable to patients with limited subtypes of localized scleroderma [33]. Nevertheless, onwards studies on human skin samples are essential [33].

### miRNA-483-5p

MicroRNA-483-5p is a molecule supposed to be a specific marker for skin fibrosis. Chouri and co-workers demonstrated that miRNA-483-5p was upregulated both in localized scleroderma (22) and systemic scleroderma (107) serum samples, unlike other autoimmunologic diseases [systemic lupus erythematosus (33), Sjögren’s syndrome (23)] [7]. These results indicate that miRNA-483-5p may be distinctive for conditions characterized by fibrosis of the skin [7]. Furthermore, researchers exhibited that miRNA-483-5p overexpression in endothelial cells increased the transcriptional levels of α-SMA (alpha-smooth muscle actin) and SM22A (smooth muscle protein 22-alpha)—indicators of myofibroblast differentiation [7]. Additionally, miRNA-483-5p decreased the level of Fli-1 (friend leukemia virus integration 1)—a negative regulator of extracellular matrix [7]. In previous studies, miRNA-483-5p was revealed to target Matn3 (matrillin 3) and TIMP2 (tissue inhibitor of metalloproteinase 2) to enhance extracellular matrix degradation, chondrocyte hypertrophy and cartilage angiogenesis [32].

## Summary

In this review, we summarized 5 clinical studies on microRNAs in localized scleroderma. The expression of miRNA-7, let 7a, 196a, 155, 483-5p was up- or downregulated, depending on their properties. Although scientists could not find any significant correlations between serum/skin microRNAs levels and clinical/laboratory findings, this may be due to small patient cohort and rarity of the disease. The delay in diagnosis and treatment of localized scleroderma may lead to uncontrolled progression of the disease and irreversible complications [18]. Therefore, miRNAs levels may be useful biomarkers of skin sclerosis severity that reflects collagen overexpression and facilitate progress towards appropriately assessing the disease.
